# Odontogenic keratocyst: An incidental finding during an orthodontic examination

**DOI:** 10.1177/1465312520924238

**Published:** 2020-06-03

**Authors:** Othman Hameed, Jamie Gwilliam, Eric Whaites

**Affiliations:** Faculty of Dentistry, Oral and Craniofacial Sciences, King’s College London, Guy’s and St Thomas NHS Foundation Trust, London, UK

**Keywords:** radiography and orthodontics (including radiation protection), imaging and cephalometry, functional appliance, odontogenic keratocyst, incidental finding, interdisciplinary treatment

## Abstract

For all orthodontic patients, a detailed clinical examination is essential to obtain the correct diagnosis before the formulation of an orthodontic treatment plan. Additional information may be required from radiographs in order to supplement this clinical examination. While orthodontists principally prescribe dental panoramic tomographs for orthodontic patients to confirm the presence, position and morphology of unerupted teeth, the absence or presence of disease within the supporting structures may be overlooked. This case report presents one such instance where the pre-orthodontic radiographic examination of a young male patient revealed the presence of an asymptomatic odontogenic keratocyst in the right posterior body of the mandible.

This case report discusses how this incidental finding was diagnosed, managed and how its surgical treatment subsequently affected the orthodontic management of the patient. Furthermore, this case highlights the importance of undertaking a thorough and systematic approach when analysing any prescribed radiographs, in order to reduce the risk of overlooking any evidence of underlying disease.

## Introduction

Clinicians have a responsibility to ensure that any requested radiograph is thoroughly and systematically assessed, with radiographic findings fully reported in the patient’s notes (Horner and Eaton, 2018). Dental panoramic tomographs (DPT) may be prescribed by orthodontists as part of the clinical assessment in order to provide an overview of both jaws and the developing dentition (Isaacson et al., 2015). This radiograph uses a relatively low dose of radiation in the range of 0.0027–0.075 mSV (Whaites and Drage, 2020). Incidental findings may be observed on these radiographs and the identification and reporting of such findings is paramount towards effective management of the patient (Vaseemuddin, 2016). This paper presents such a case, where an incidental finding of an odontogenic keratocyst (OKC) was discovered during an orthodontic new patient examination. This paper discusses how this incidental finding was diagnosed and managed, and how its treatment subsequently affected the orthodontic management of the patient.

## Case report

### History

A 12-year-old male patient was referred to the orthodontic department at King’s College Hospital by an orthodontic specialist for an opinion regarding his increased overjet. The patient’s presenting complaint related to the appearance of his upper anterior teeth, which he felt ‘stick out too much’. There was no relevant medical or social history.

### Clinical examination

**Figure 1.**
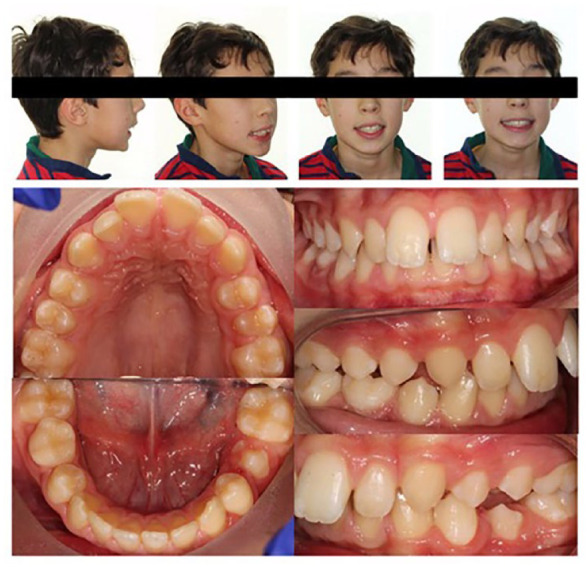
Pre-treatment extra-oral and intra-oral photographs.

Extra-orally, the patient presented with a moderate skeletal II base, with average vertical proportions and no obvious transverse asymmetries. His lips were incompetent at rest with the presence of a lower lip trap. Intra-orally, the patient was in the late mixed dentition with his lower right second deciduous molar being the only primary tooth that remained. His oral hygiene was excellent, with healthy gingivae and a non-restored dentition. Both the upper and lower dentitions were well aligned with evidence of generalised spacing. In occlusion, he presented with bilateral ½ unit Class II molar and canine relationships, a Class II division I incisor relationship with an increased overjet of 8 mm and an increased overbite which was complete to tooth (Figure 1).

### Radiographic investigation

**Figure 2.**
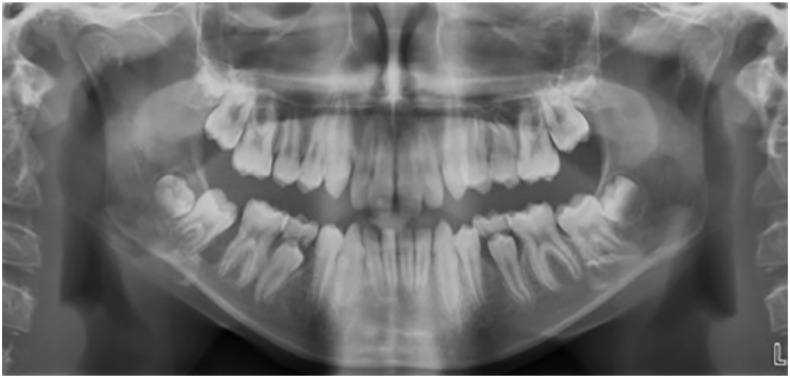
Pre-treatment DPT showing a radiolucency between the LR6 and unerupted LR7.

**Figure 3.**
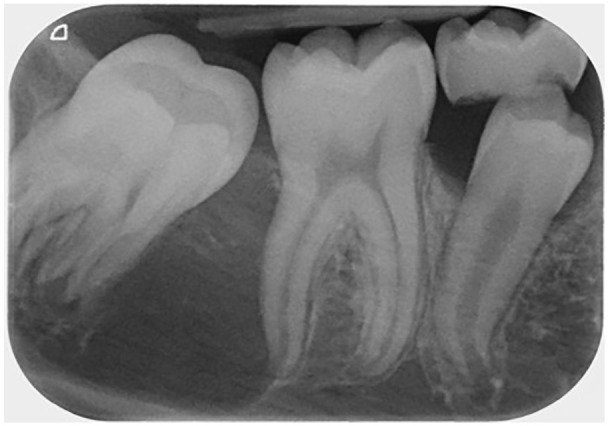
Long cone periapical radiograph of the LR6/LR7 region confirming the presence of a lesion.

Initially, both a true lateral cephalometric skull radiograph and a DPT were taken in order to assist with diagnosis and treatment planning. The cephalometric analysis supported the clinical findings, particularly the soft tissue and skeletal relationships. The DPT confirmed that all adult teeth were present, but showed a radiolucency situated between the lower right first molar and unerupted lower right second molar tooth (Figure 2). A periapical radiograph was taken to further assess this potential lesion (Figure 3). This radiograph confirmed the presence of what appeared to be a well-defined, uniformly radiolucent, unilocular lesion extending from the distal surface of the lower right first molar towards the apical region of the lower right second molar measuring approximately 5 × 10 mm in size.

### Further investigations

This incidental finding prompted further clinical examination. The lower right first molar was neither displaced nor mobile. In addition, the tooth exhibited positive results to vitality testing, ruling out a radicular cyst as a differential diagnosis. The right posterior mandible was rechecked intra-orally and the absence of any swelling or fluctuance within this region was confirmed. After discussion with an on-site radiologist, the following differential diagnoses were considered:

### Differential diagnoses

*Odontogenic keratocyst*: These rare lesions account for around 5% of all jaw cysts (Odell, 2017). They arise from the epithelium of the dental lamina and can occur within any part of the jaw but are most commonly seen in the posterior body of the mandible (90% occur posterior to the canines) and ramus (Mallya and Lam, 2018). Although they are classified as benign developmental cysts, they can exhibit a potentially aggressive infiltrative behaviour. They appear as well defined, uniformly radiolucent lesions which can be either unilocular or multilocular. As they tend to be symptomless, rarely causing displacement or resorption of adjacent teeth, and expansion is uncommon, these lesions are often undetected and can be very large when discovered radiographically. They are often radiographically indistinguishable from a dentigerous cyst, as they can also situate in a similar pericoronal position.

*Dentigerous cyst*: These are a common cause of large radiolucent lesions at the posterior of the mandible and are associated with the cervical area of the crown of an unerupted and displaced tooth. Although they account for 20% of all odontogenic cysts, they are uncommon in children and are most frequently found between the ages of 20 and 50 years (Table 1).

**Table 1.** Relative frequency of different types of odontogenic cysts.

Radicular cyst	65%
Dentigerous cyst	24%
Odontogenic keratocyst	5%
Lateral periodontal cyst	1%
Paradental cyst	<1%

*Lateral periodontal cyst*: These rare developmental cysts develop in the lateral periodontal region of vital teeth and are typically unilocular, well-defined and uniformly radiolucent. However, these lesions are most often found at either the lateral surface of the lower canine/premolar teeth or upper lateral incisor region.

*Solitary bone cyst*: These non-neoplastic osseous lesions are also referred to as ‘traumatic bone cyst’ or ‘simple bone cyst’ and comprise 1% of all cysts affecting the jaws (Wright and Vered, 2017). They are generally asymptomatic, incidental findings most commonly found in the mandibular body between the canine and third molar teeth (Madiraju et al., 2014). Although they are relatively uncommon lesions, they do present more often in adolescent patients and therefore should be strongly considered as a differential diagnosis for this 12-year-old patient.

*Odontogenic tumour*: The main odontogenic tumours which affect children include ameloblastic fibromas and ameloblastomas. However, these benign tumours are still extremely rare. As they grow, these lesions are likely to cause gross expansion, displace adjacent teeth and can sometimes lead to root resorption. None of these features were present upon examination of this particular patient.

Despite all these differential diagnoses being considered, definitive diagnosis of a lesion is normally only confirmed following review of a biopsy sample.

### Surgical management

**Figure 4.**
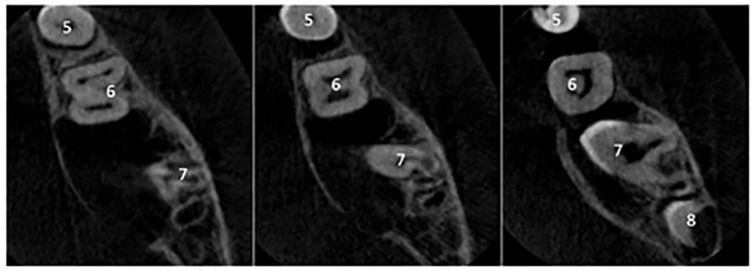
Axial views of the CBCT of the right mandible. These images show the full extent of the lesion, which appears to be pseudolocular, attached to the cementoenamel junction of the LR7 and causing obvious bony expansion towards the lingual surface. Although no obvious root resorption is seen on the LR7, there appears to be superior displacement of its roots with resultant lingual rolling of the crown.

**Figure 5.**
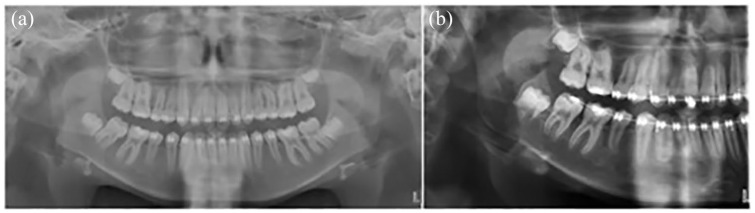
(a) DPT taken six months after removal of the OKC showing good bony infill and (b) sectional DPT taken 12 months after surgery showing no signs of recurrence.

- The patient was referred to the oral surgery department who requested a cone beam computed tomography (CBCT) scan to assess the area in further detail (Figure 4). The CBCT scan showed the full extent of the lesion, which had caused bony expansion and displacement of the lower right second molar. After this scan, the decision was made to enucleate the lesion under general anaesthetic. Due to the possibility that this lesion was an OKC, the cyst cavity was treated with Carnoy’s solution after removal of the cyst lining. This was because OKCs have a high propensity for reoccurrence, which is thought to be due to small satellite cells or fragments of epithelium left behind after surgical removal of the main cyst (Mallya and Lam, 2018). Carnoy’s solution is thought to reduce the risk of reoccurrence following enucleation due its ability to penetrate cancellous spaces within the bone and its fixation action upon any potential residual cystic cells (Kaczmarzyk et al., 2012).
- A specimen was sent for histopathological examination which confirmed the diagnosis of an OKC.
- A postoperative DPT was taken six months after the surgery and a sectional DPT was taken after a further period of six months. These images showed good bony infill at the operation site with no signs of reoccurrence (Figure 5).
- However, the patient is being followed up in the long term by the oral surgery department due to the high reoccurrence rate of OKCs, which has been reported as in the range of 16%–30% (Bande et al., 2010).

### Orthodontic management

**Figure 6.**
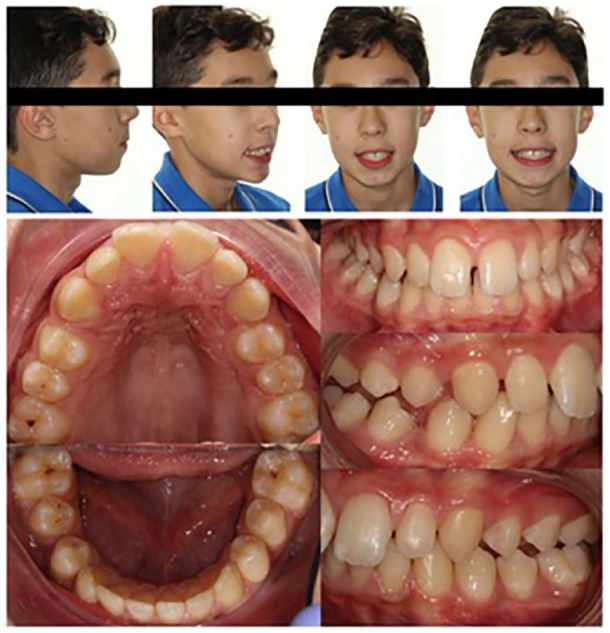
Post-functional appliance treatment extra-oral and intra-oral photographs.

**Figure 7.**
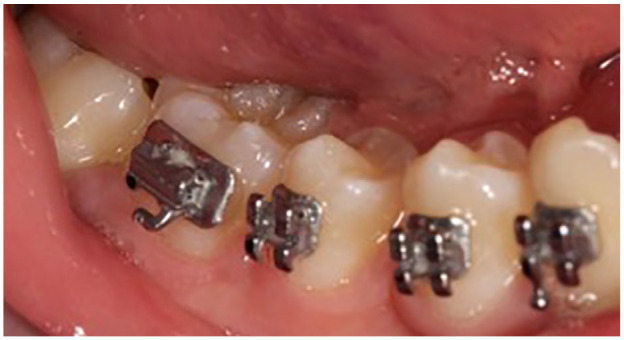
Intra-oral photograph showing successful eruption of the LR7 following removal of the OKC. Note the tooth appears lingually inclined. It is uncertain whether this is due to previous displacement from the OKC.

**Figure 8.**
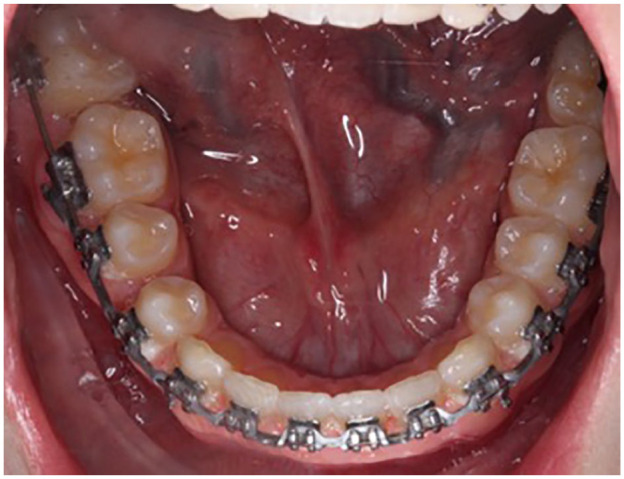
Near end of treatment lower occlusal intra-oral photograph showing successful alignment of the LR7 after treatment with fixed appliances.

- Concurrently, the patient was treated by the orthodontic department regarding his Class II malocclusion. Initially, he was treated with a modified Clark Twin Block appliance in order to enable correction of the increased overjet, overbite and Class II buccal segment relationships. Progression with a removable functional appliance was considered appropriate for this case as the patient was able to remove the appliance for his surgical procedure, while being able to continue full time wear during the period of bony infill of the cystic region.
- After nine months of full-time wear of the modified Clark Twin Block appliance, the objectives of the first stage of orthodontic treatment had been achieved (Figure 6). The patient was advised to continue to wear the functional appliance at night-time only for three months in order to allow for resolution of lateral open bites and maintain the anteroposterior improvement achieved.
- Following this period of transition and post-surgical healing, the patient was treated on a non-extraction basis, with fixed upper and lower pre-adjusted edgewise appliances (0.022 × 0.028-inch slot, MBT prescription) to align and detail the occlusion while maintaining the overjet and overbite correction. Initially, the lower right second molar was excluded from the fixed appliances as it was only partially erupted and confirmation of uneventful healing of the surgical site from the oral surgery department was necessary. Over time, the lower right second molar erupted further, however with a lingual inclination (Figure 7). Once the oral surgery department had confirmed uneventful healing of the surgical site, the lower right second molar was bonded and aligned with the fixed appliances. As the lower right second molar erupted with a lingual inclination and the overbite was still relatively deep, anterior bite turbos were added to the palatal surface of the upper central incisors to disclude the dentition, allowing alignment of the lower right second molar and further reduction of the deep overbite (Figure 8).
- After the completion of fixed appliance treatment, upper and lower vacuum formed retainers were provided to retain the achieved occlusion.

## Discussion

Although DPTs are primarily used for orthodontic patients to confirm the presence, position and morphology of unerupted teeth, it is important to remember that they also provide an overview of both jaws. For this reason, a thorough and systematic approach is necessary when analysing these radiographs in order to reduce the risk of overlooking evidence of underlying disease.

Management of this OKC required a multidisciplinary approach utilising both the radiology and oral surgery departments. Although every attempt was made to successfully remove the OKC by the oral surgery department, there remains no absolute certainty of complete removal due to its high recurrence rate. Most recurrences occur within the first 5–7 years after treatment; however, some reports have shown recurrences appearing up to 40 years after enucleation (Woolgar et al., 1987). For these reasons, annual radiographic monitoring is recommended for the first five years after removal of the OKC, with subsequent follow-up at least every two or three years (Scarfe et al., 2018).

The orthodontic management of the patient was complicated by this diagnosis. Initially, the long term-prognosis of both the lower right first and second molars was unknown due to their proximity to the OKC. Fortunately, following successful removal of the OKC, both these teeth remained unharmed and now have a favourable long-term prognosis. However, it was still necessary to allow a period of healing and bony infill and repair following removal of the OKC, before inclusion of the lower right second molar in the fixed appliances. Inclusion and uprighting of the lower right second molar were also complicated as this tooth was lingually tipped. It remains uncertain whether the lingual inclination of the tooth upon eruption was due to displacement by the OKC because, as previously mentioned, OKCs rarely cause displacement of adjacent teeth.

## Conclusion

This case highlights the importance of thoroughly assessing all available radiographs and of seeking a second opinion where uncertain. Through a multidispliclinary approach this patient has been safely managed and continues to be reviewed at hospital by both the orthodontic and oral surgery departments.
